# Association between vitamin C intake and the risk of pancreatic cancer: a meta-analysis of observational studies

**DOI:** 10.1038/srep13973

**Published:** 2015-09-11

**Authors:** Hua Fan, Jiantao Kou, Dongdong Han, Ping Li, Dong Zhang, Qiao Wu, Qiang He

**Affiliations:** 1 Department of Hepatobiliary Surgery, Beijing Chaoyang Hospital, Capital Medical University, Beijing, China

## Abstract

Quantification of the association between the intake of vitamin C and risk of pancreatic cancer is still conflicting. We therefore conducted a meta-analysis to assess the association between them. Pertinent studies were identified by a search of PubMed and Web of Knowledge throughSeptember of 2014. A random effects model was used to combine the data for analysis. Sensitivity analysis and publication bias were conducted. Data from 17 studies including 4827 pancreatic cancer cases were used in this meta-analysis. Pooled results suggested that highest vitamin C intake amount versus lowest amount was significantlyassociated with reduced the risk of pancreatic cancer [summary relative risk (RR) = 0.705, 95% CI = 0.612–0.811, I^2^ = 42.3%]. The associations were also significant both in Caucasian [summary RR = 0.741, 95% CI = 0.626–0.876], Asian [summary RR = 0.455, 95% CI = 0.275–0.754] and Mixed population [summary RR = 0.677, 95% CI = 0.508–0.901]. No publication bias was found. Our analysis suggested that the higher intake of vitamin C might reduce the risk of pancreatic cancer.

Pancreatic cancer is the eighth most common cause of cancer death in Europe^1^ and the fourth in the United States^2^. Because pancreatic cancer is most often diagnosed at a late stage, prognosis is poor with 1-year survival rates of 20% and 5-year survival rates of only 4–5%^2^,^3^. Several risk factors have been consistently associated with the risk of developing pancreatic cancer, including family history of pancreatic cancer^4^, chronic pancreatitis^5^, cigarette smoking^6^, diabetes mellitus^7^ and obesity^8^.

Diet may be involved in the aetiology of pancreatic cancer and dietary variations between countries may explain the differences in incidence. For antioxidants, such as vitamins C, there are several plausible biological mechanisms by which they might prevent pancreatic cancer, including inactivating free radicals and reducing oxidative DNA damage, stimulating immune function^9^ and through genetic effects^10^. Up to date, a number of epidemiologic studies have been published to explore the relationshipbetween vitamins C intake and pancreatic cancer risk. Some studies reported that higher vitamin C intake could reduce the pancreatic cancer risk^11^,^12^,^13^,^14^,^15^,^16^,^17^,^18^, while some other studies reported that vitamin C intake had nonsignificant association with the risk of pancreatic cancer^19^,^20^,^21^,^22^,^23^,^24^,^25^,^26^,^27^. Therefore, we conducted a meta-analysis to (1) assesspancreatic cancer risk in subjects with highest and lowest reported values of vitamins C intake; (2) assess heterogeneity and publication bias among the studies we analyzed.

## Methods

### Search Strategy

Studies were identified using a literature search of PubMed and Web of Knowledge through September 2014 and by hand-searching the reference lists of the retrieved articles. The following search terms were used: ‘pancreatic cancer’ or ‘pancreatic carcinoma’ combined with ‘nutrition,’ ‘diet,’ ‘lifestyle,’ ‘vitamin C,’‘vitamins’ or ‘ascorbic acid’. Two investigators searched articles and reviewed all the retrieved studies independently. Disagreements between the two investigators were resolved by consensus with a third reviewer.

### Study Selection

For inclusion, studies had to fulfill the following criteria: (1) have a prospective or case-control or retrospective cohort studies; (2) vitamin C intake was the independent variable of interest; (3) the dependent variable of interest was pancreatic cancer; (4) relative risk (RR) or odds ratio (OR) or hazardratio (HR) with a 95% confidence interval (CI) was provided (we presented all results with RR for simplicity). If data were replicated in more than one study, we included the study with the largest number of cases. Accordingly, the following exclusion criteria were also used: (1) reviews; (2) repeated or overlapped publications.

### Data extraction

Two researchers independently extracted the following data from each study that met the criteria for inclusion: the first author’s last name, year of publication, geographic locations, study design, sample source, the age range of study participants, duration of follow-up, the number of cases and participants, and RR (95% CI) for vitamin C intake and pancreatic cancer risk. From each study, we extracted the RR that reflected the greatest degree of control for potential confounders. If there was disagreement between the two investigators about eligibility of the data, it was resolved by consensus with a third reviewer.

### Quality assessment

To determine the quality score of included studies, two reviewersindependently performed the quality assessment by using the Newcastle-Ottawa Scale^28^, which is a validated scale for non-randomized studiesin meta-analyses^29^. The Newcastle-Ottawa Scale is anine-point scale that allocates points based on the selection process of cohorts (0–4 points), the comparability of cohorts (0–2 points), and the identification of the exposure and the outcomes of study participants (0–3 points). We assigned scores of 0–3, 4–6, and 7–9 for low, moderate, and high quality of studies, respectively.

### Statistical analysis

The pooled measure was calculated as the inverse variance-weighted mean of the logarithm of RR with 95% CI, to assess the association between vitamin C intake and pancreatic cancer risk. Random-effects model was used to combine study-specific RR (95% CI), which considers both within-study and between-study variation^30^. The I^2^ was used to assess heterogeneity, and I^2^ values of 0, 25, 50 and 75% represent no, low, moderate and high heterogeneity^31^, respectively. Meta-regression with restricted maximum likelihood estimation was performed to assess the potentially important covariates that might exert substantial impact on between-study heterogeneity^32^. Publication bias was evaluated using Egger’s regression asymmetry test^33^. The Duval and Tweedie nonparametric trim-and-fill method was performed to further assess the potential publication bias^34^. A study of influence analysis^35^ was conducted to describe how robust the pooled estimator was to removal of individual studies. An individual study was suspected of excessive influence if the point estimate of its omitted analysis lay outside the 95% CI of the combined analysis. All statistical analyses were conducted with STATA version 11.0 (StataCorp LP, College Station, Texas, USA). Two-tailed p-value ≤ 0.05 was accepted as statistically significant.

## Results

### Search results and study characteristics

**Figure 1:**
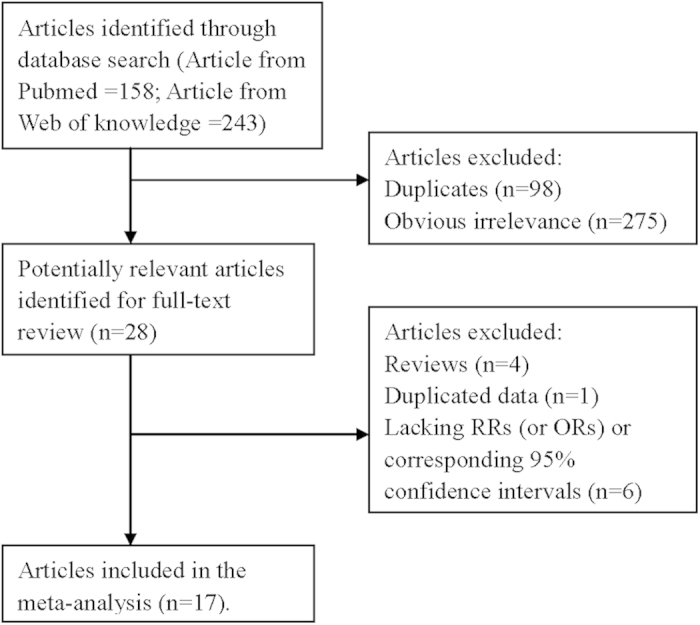
The flow diagram of screened, excluded, and analyzed publications.

The search strategy identified 158 articles from PubMed and 243 from the Web of Knowledge, and 28 articles were reviewed in full after reviewing the title/abstract. In total, 17 articles^11^,^12^,^13^,^14^,^15^,^16^,^17^,^18^,^19^,^20^,^21^,^22^,^23^,^24^,^25^,^26^,^27^ (4 cohort studies and 13 case-control studies) involving 4827 pancreatic cancer cases were used in this meta-analysis after reviewed in full articles. The detailed steps of our literature search are shown in Fig. 1. Four studies were conducted in the United States, 2 in the Canada, 9 in the Europe, 1 in the Japan and 1 in the Australia. The characteristics of these studies are presented in Table 1. The quality of studies was generally good, with results of study quality assessment yielded a score of 6 or above for all included studies, with an average score of 7.2.

### High versus low analyses

**Figure 2:**
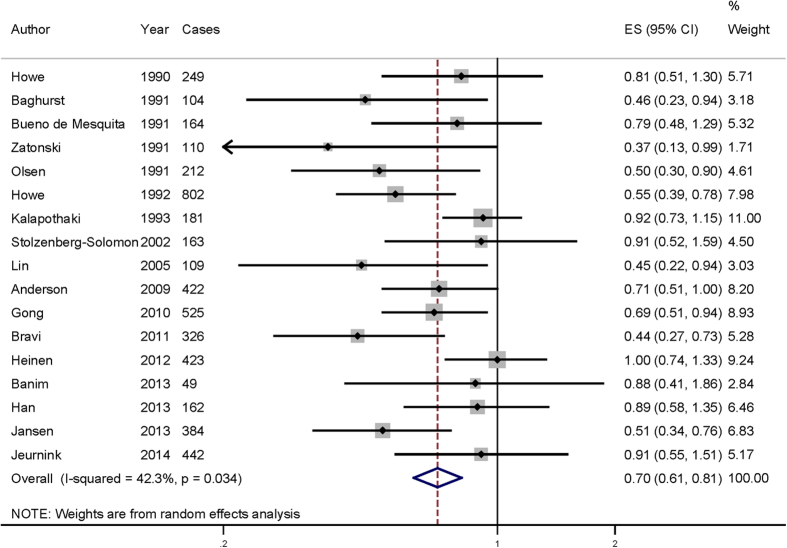
The forest plot between highest versus lowest categories of vitamin C intake and pancreatic cancer risk.

Eight of the studies included in our analysis reported an inverse association of vitamin C intake with the risk of pancreatic cancer, while no significant association was reported in 9 studies. Our pooled results suggested that the highest vitamin C intake amount compared to the lowest amount was significantly associated with the risk of pancreatic cancer [summary RR = 0.705, 95% CI = 0.612–0.811, I^2^ = 42.3%] (Fig. 2).

When the studies were stratified bystudy design, the associations were also found in the case-control studies [summary RR = 0.648, 95% CI = 0.553–0.760] and in the cohort studies [summary RR = 0.827, 95% CI = 0.651–0.994]. For subgroup analyses of ethnicity, we divided into Caucasian, Asian and Mixed population (one study from United States was Caucasian and the other three United States were Mixed population). Highest vitamin C intake level versus lowest level was significantly associated with the risk of pancreatic cancer both in Caucasian [summary RR = 0.741, 95% CI = 0.626–0.876], Asian [summary RR = 0.455, 95% CI = 0.275–0.754] and Mixed population [summary RR = 0.677, 95% CI = 0.508–0.901]. The detailed results are summarized in Table 2.

### Sources of heterogeneity and meta-regression

As shown in the pooled results, moderate heterogeneity (I^2^ = 42.3%, *P*_heterogeneity_ = 0.034) was found in the analysis. In order to explore the moderate to high between-study heterogeneity founded in several analysis, univariate meta-regression with the covariates of publication year, ethnicity, study design (case-control or prospective), number of cases and source of controls was performed. No significant findings were found in the above-mentioned analysis. Considering the adjustment of individual studies is heterogeneous, we then provide the original unadjusted relative risks and pooled them together to derive an effect size estimate. The pooled RR was 0.771 (95% CI = 0.685–0.868) for vitamin C intake and pancreatic cancer risk. Low heterogeneity was found (I^2^ = 12.1%, *P*_heterogeneity_ = 0.331).

### Influence analysis and publication bias

**Figure 3:**
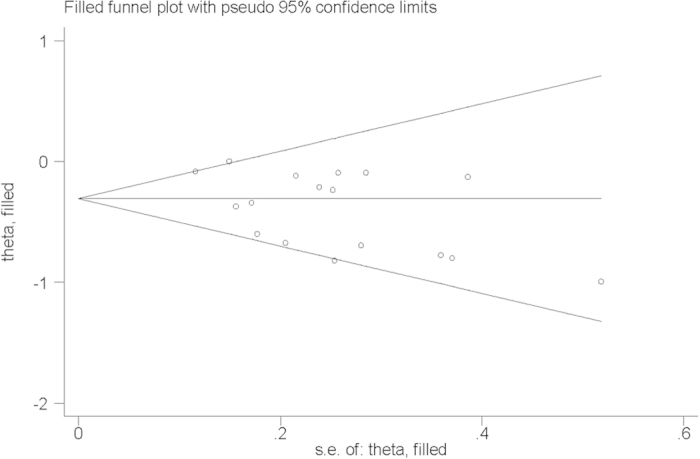
Filled funnel of relative risk of studies that investigated the association between vitamin C intake and pancreatic cancer risk.

Influence analysis showed that no individual study had excessive influence on the association of vitamin C intake and pancreatic cancer risk. The trim-and-fill funnels (Fig. 3) and Egger’s test (*P* = 0.414) showed no evidence of significant publication bias between vitamin C intake and pancreatic cancer risk.

## Discussion

Finding from this meta-analysis suggested that the higher intake of vitamin C could reduce the risk of pancreatic cancer. The associations were also found in subgroups of Caucasian, Asian and Mixed population for vitamin C intake and pancreatic cancer risk.

Vitamin C is one of the most common antioxidants in fruits and vegetables, and it may exert chemopreventive effects^36^. It has generally been acknowledged that vitamin C protects cells from oxidative DNA damage, thereby blocking carcinogenesis^37^. A second mechanism for antioxidants is their effect on the inflammatory process, and chronic inflammation may play a role pancreatic carcinogenesis^38^. The results of our analysis had verified this hypothesis.

Munafo and Flint reported that between-study heterogeneityis common in meta-analyses^39^. Exploring potential sources of between-study heterogeneity is therefore an essential component of meta-analysis. We found a moderate degree of heterogeneity (I^2^ = 42.3%, *P*_heterogeneity_ = 0.034) in our pooled results. This might havearisen from publication year, study design, geographic location, and sources of controls or number of cases. Thus, we used meta-regression to explore the causes of heterogeneity for covariates. However, no covariate having a significant impact on between-studyheterogeneity was found among those mentioned above. We then performed subgroup analyses by the type of study design (prospective or case-control studies) and ethnicityto explore the sourceof heterogeneity. However, between-study heterogeneity persisted insome of the subgroups, suggesting the presence of other unknown confounding factors. Considering the adjustment of individual studies is heterogeneous, we then provide the original unadjusted relative risks and pooled them together to derive an effect size estimate. The pooled RR was 0.771 (95% CI = 0.685–0.868) for vitamin C intake and pancreatic cancer risk. Low heterogeneity was found (I^2^ = 12.1%, *P*_heterogeneity_ = 0.331). This may be because the adjustment of individualstudies for confounding factors is different.

As a meta-analysis of published studies, our findings showed some advantages. First, this is the first comprehensivemeta-analysis of vitamin C intake and pancreatic cancer risk based on highestamountversus lowest amount analysis. Second, large number of cases and participants was included, allowing a much greater possibility of reaching reasonable conclusions between vitamin C intake and pancreatic cancer risk. Third, no significant publication bias was found, indicating that our results are stable.

There were also some limitations in this meta-analysis. First, a meta-analysis of observationalstudies is susceptible to potential bias inherent in the original studies, especially for case-control studies. Overstated association may be expected from the case-control studies because of recall or selection bias. Although case-control studycan allow a recall or selection bias, case-control studyis an important method in etiology research. In order to find whether the study design is a key contributor to the between-study heterogeneity, univariate meta-regression with study design (case-controlor cohort) was performed. No significant finding (*P* = 0.39) was found in the above-mentioned analysis. However, significant associations were found both in case-controls studies and in cohort studies. More studies with prospective design are wanted in the future studies while only 4 studies included in this meta-analysis were prospective design. Second, measurement errors are important in the assessment of dietary intake, which can lead to overestimation of the range of intake and underestimation of the magnitude of the relationship between dietary intake and cancer risk^40^,^41^. Third, in our meta-analysis, we used ‘highest versus lowest vitamin C intake’. Although some references use quartiles and some use quintiles to partition vitamin C intake considering that differing definitions can be a source of heterogeneity, Egger’s test (*P* = 0.414) showed no evidence of significant publication bias was found suggesting that our results are stable. Fourth, there seems to be a big gap in published material between 1993 and 2002 in our meta-analysis. For this reason, we have searched the databasecarefully and by hand-searching the reference lists of the retrieved articles again, and did not find any related articles. Fifth, there appears to be a large variability in the baseline riskdue to the limitation of published material used, for whichwe cannot change. Finally, between-study heterogeneity was found in the pooled analysis, and the between-study heterogeneity was successfully explained by themeta-regression.

In summary, results from this meta-analysis suggested that the higher intake of vitamin C might reduce the risk of pancreatic cancer.

## Additional Information

**How to cite this article**: Fan, H. *et al.* Association between vitamin C intake and the risk of pancreatic cancer: a meta-analysis of observational studies. *Sci. Rep.* **5**, 13973; doi: 10.1038/srep13973 (2015).

## Figures and Tables

**Table 1:** Characteristics of studies on vitamin C intake and pancreatic cancer risk.

Study, year	Country	Study design	Participants (cases)	Age (years)	RR (95% CI) for highest versus lowest category	Adjustment for covariates
Howe *et al.* 1990	Canada	Case-control	754(249)	35–79	0.81(0.51–1.30)	Adjust for caloric and fibre intake, lifetime cigarette consumption.
Baghurst *et al.* 1991	Australia	Case-control	357(104)	<50-≥80	0.46(0.23–0.94)	Adjust for age; pack-years of smoking, tobacco consumption and viceversa.
Bueno de Mesquita *et al.* 1991	Netherlands	Case-control	644(164)	35–79	0.79(0.48–1.29)	Adjust for age, sex, response status, total smoking and dietary intake of energy.
Zatonski *et al.* 1991	Poland	Case-control	305(110)	62.2	0.37(0.13–0.99)	Adjust for cigarette lifetime consumption and calories.
Olsen *et al.* 1991	United States	Case-control	432(212)	40–84	0.5(0.3–0.9)	Adjusted for total energy, age, cigarette usage, alcohol consumption, respondent-reported history of diabetes mellitus, and educational level.
Howe *et al.* 1992	Europe	Case-control	2471(802)	28–87	0.55(0.39–0.78)	Adjusted for age, sex, nutrient variables (categorical), and lifetime cigarette consumption (continuous).
Kalapothaki *et al.* 1993	Greece	Case-control	362(181)	*Na*	0.92(0.73–1.15)	Adjust for age, gender, hospital, pastresidence, years of schooling, cigarette smoking, diabetes mellitus and energy intake.
Stolzenberg-Solomon *et al.* 2002	Finland	Prospective	27111(163)	50–69	0.91(0.52–1.59)	Adjust for by the residual method and for age and years of smoking, energy-adjusted folate intake and energy-adjusted saturated fat intake.
Lin *et al.* 2005	Japan	Case-control	327(109)	40–79	0.45(0.22–0.94)	Adjust for age, pack-years of smoking and energy intake.
Anderson *et al.* 2009	Canada	Case-control	734(422)	<79	0.71(0.51–1.00)	Age-adjusted odds ratio. Age at pancreas cancer diagnosis date for cases and at referent date of 1 January 2003 (midpoint of caserecruitment) for controls.
Gong *et al.* 2010	United States	Case-control	2226(525)	21–85	0.69(0.51–0.94)	Adjusted for age in 5-year groups, sex and total energy intake, race, education, body mass index, history of diabetes, smoking, physical activity, and alcoholconsumption.
Bravi *et al.* 2011	Italian	Case-control	978(326)	34–80	0.44(0.27–0.73)	Adjusted for age, sex, and center, year of interview, education, tobacco smoking, and history of diabetes, body mass index, and total energy intake.
Heinen *et al.* 2012	Netherlands	Prospective	120825(423)	55–69	1.00(0.74–1.33)	Adjusted for age, sex, smoking, body mass index, familyhistory of pancreatic cancer, history of diabetes mellitus, intake of energy, red meat, coffee, and alcohol.
Banim *et al.* 2013	UK	Prospective	23658(49)	40–74	0.88(0.41–1.86)	Adjusted for age, sex, smoking, diabetes, total energy intake and body mass index category.
Han *et al.* 2013	United States	Prospective	77446(162)	50–76	0.89(0.58–1.35)	Adjusted for age, gender, ethnicity, education, body mass index, physical activity, cigarette smoking status, total alcohol consumption, family history of pancreatic cancer, history of diabetesand total energy intake.
Jansen *et al.* 2013	United States	Case-control	1367(983)	31–92	0.51(0.34–0.76)	Adjusted for energy, smoking, BMI, age, sex, and drinks of alcohol per week
Jeurnink *et al.* 2014	Europe	Nested case-control	521468(442)	52.1	0.91(0.55–1.51)	Adjusted for age at blood collection, study center, sex, date of blood collection, time of blood collection, fasting status and hormone use, smoking status, duration and intensity of smoking, cotinine levels, waist circumference and diabetes status..

**Table 2:** Summary risk estimates of the association between vitamin C intake and pancreatic cancer risk.

Subgroups	No. (cases)	No. studies	Risk estimate (95% CI)	Heterogeneity test I^2^ (%) P-value	
All studies	4827	17	0.705(0.612–0.811)	42.3	0.034
Study design					
Prospective	797	4	0.827(0.651–0.994)	0.0	0.965
Case-control	4030	13	0.648(0.553–0.760)	41.1	0.060
Ethnicity					
Caucasian	3543	12	0.741(0.626–0.876)	43.4	0.054
Asian	213	2	0.455(0.275–0.754)	0.0	0.966
Mixed	1071	3	0.677(0.508–0.901)	43.4	0.171
